# Randomized controlled study using text messages to help connect new medicaid beneficiaries to primary care

**DOI:** 10.1038/s41746-021-00389-5

**Published:** 2021-02-15

**Authors:** David M. Levine, Pragya Kakani, Ateev Mehrotra

**Affiliations:** 1 Division of General Internal Medicine and Primary Care, Brigham and Women’s Hospital, Boston, MA USA; 2 Harvard Medical School, Boston, MA USA; 3 Department of Health Care Policy, Harvard Medical School, Boston, MA USA; 4 Division of General Medicine, Beth Israel Deaconess Medical Center, Boston, MA USA

**Keywords:** Health policy, Patient education

## Abstract

Accessing primary care is often difficult for newly insured Medicaid beneficiaries. Tailored text messages may help patients navigate the health system and initiate care with a primary care physician. We conducted a randomized controlled trial of tailored text messages with newly enrolled Medicaid managed care beneficiaries. Text messages included education about the importance of primary care, reminders to obtain an appointment, and resources to help schedule an appointment. Within 120 days of enrollment, we examined completion of at least one primary care visit and use of the emergency department. Within 1 year of enrollment, we examined diagnosis of a chronic disease, receipt of preventive care, and use of the emergency department. 8432 beneficiaries (4201 texting group; 4231 control group) were randomized; mean age was 37 years and 24% were White. In the texting group, 31% engaged with text messages. In the texting vs control group after 120 days, there were no differences in having one or more primary care visits (44.9% vs. 45.2%; difference, −0.27%; *p* = 0.802) or emergency department use (16.2% vs. 16.0%; difference, 0.23%; *p* = 0.771). After 1 year, there were no differences in diagnosis of a chronic disease (29.0% vs. 27.8%; difference, 1.2%; *p* = 0.213) or appropriate preventive care (for example, diabetes screening: 14.1% vs. 13.4%; difference, 0.69%; *p* = 0.357), but emergency department use (32.7% vs. 30.2%; difference, 2.5%; *p* = 0.014) was greater in the texting group. Tailored text messages were ineffective in helping new Medicaid beneficiaries visit primary care within 120 days.

## Introduction

Primary care use—care that is first-contact, comprehensive, coordinated, and continuous^1,2^—is associated with better quality, better care experience, and increased life expectancy^3,4^. Engaging with a new primary care provider (PCP) can be difficult, particularly for patients with Medicaid^5,6^. A key moment of opportunity, but also of confusion, occurs when beneficiaries enroll in a new plan. New beneficiaries may not understand which PCPs are in the plan’s network or how to contact an assigned PCP^7^. Compared to those with commercial insurance, Medicaid enrollees are less likely to have a PCP over time^8^ and in some communities few PCPs accept Medicaid^9^. Studies of Medicaid expansion have found that there are increases in emergency department (ED) use^10,11^, which is attributed partly to the inability for new enrollees to engage with primary care. Helping new Medicaid beneficiaries obtain a PCP remains a major challenge^5,12^.

One potential intervention to increase PCP use is text messages. In prior work, though the effects are not always consistent, text messages decreased no-show rates in clinics^13–16^, including in underserved populations^17,18^. Given 95% of low-income Americans own a cell phone (71% own a smartphone), text messages are becoming ubiquitous and therefore may be a lower-cost, more convenient, and more effective mechanism to reach this underserved population over phone calls or mail^19^. A new development is the use of automated tailored text messages in which a computer uses an algorithm to respond to typical questions or responses from patients. This allows for a relatively lower cost, but still a “higher touch” and personalized experience. Whether tailored text messages can help newly enrolled patients engage with primary care within 120 days after enrollment is unknown.

In a randomized control trial we test the effect of tailored text messages on visiting primary care within 120 days of enrollment into a Medicaid plan.

## Results

### Participant characteristics

**Fig. 1:**
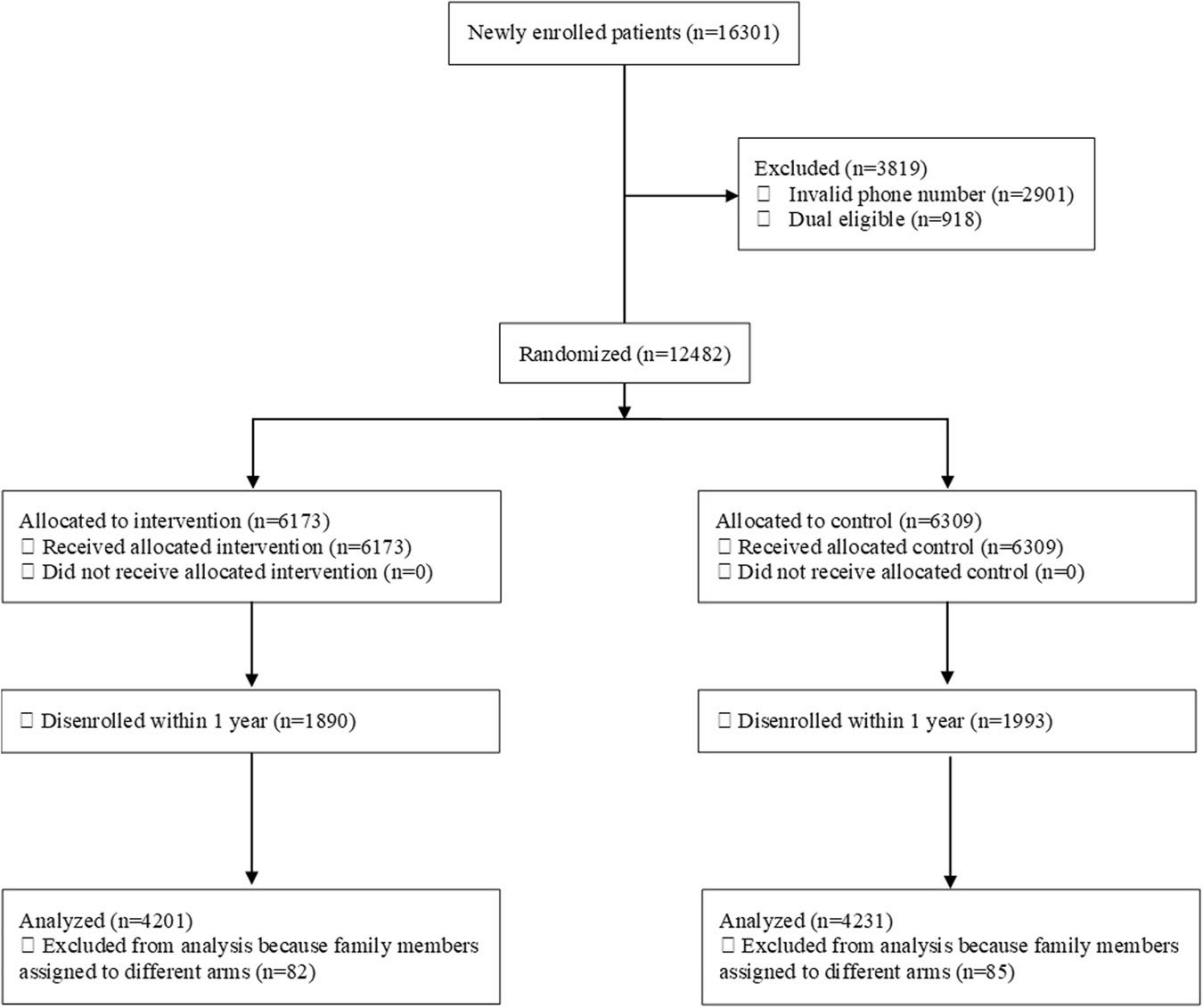
Participant flow. The Consolidated Standards of Reporting Trials flow diagram.

After our exclusion criteria there were 8432 newly enrolled beneficiaries randomized (4201 texting group; 4231 control group; Fig. 1 and Table 1). Over half (55.8%) were female with a mean age of 37.0 years. Most (89.0%) spoke English. Many (47.3%) were Hispanic, while 23.9% were White, 10.3% were Black, and 10.8% were of unknown race/ethnicity. There were no meaningful differences between the two groups at baseline on the measured characteristics. Within 1 year, 30.7% of the texting group and 31.7% of the control group had disenrolled from the plan (difference, −1.0%; 95% CI, −2.7% to 0.6%; *p* = 0.22).

**Table 1:** Baseline participant characteristics.

	Control (*n* = 4231)	Texting (*n* = 4201)
Age, mean (95% CI)	36.9 (36.5, 37.3)	37.1 (36.7, 37.6)
Female, *n* (%)^a^	2374 (56.1%)	2326 (55.4%)
English is first language, *n* (%)	3736 (89.0%)	3741 (89.1%)
*Race/ethnicity, n (%)*		
White	1005 (23.7%)	1007 (24.0%)
Black	449 (10.6%)	417 (9.9%)
Hispanic	1993 (47.1%)	1996 (47.5%)
Other	328 (7.7%)	325 (7.7%)
Unknown	456 (10.8%)	456 (10.8%)

^a^Three individuals were missing data on gender.

Of the 4201 beneficiaries in the texting group, 2116 (50.4%) replied in some form to the initial text message, 1305 beneficiaries (31.1%) responded with a response other than “stop”, and 432 beneficiaries (10.3%) responded more than four times with a response other than stop. Beneficiaries in the texting arm who engaged with the texts (i.e., responded with a response other than “stop”) were older, more likely to be female, and less likely to be Hispanic than those in the texting arm who did not engage with the texts (Supplementary Table 2).

### Engagement with primary care within 120 days

At 120 days, 44.9% of the texting group and 45.2% of the control group had one or more primary care visits (difference, −0.27%; 95% CI, −0.94 to 0.41%; *p* = 0.44; Table 2). Completion of the IHA was also similar: 44.8% in the texting group and 45.0% in the control group (difference, −0.25%; 95% CI, −2.37 to 1.87%; *p* = 0.82).

**Table 2:** Text message outcomes.

	Control (*n* = 4231)	Texting (*n* = 4201)	Intention-to-treat analysis		Instrumental variable analysis	
	*n* (%)	*n* (%)	Difference (%)	*p*-value	Difference (%)	*p*-value
*Outcomes captured at 120 days*						
Primary care visit	1913 (45.2)	1888 (44.9)	−0.27	0.80	−0.88	0.80
IHA completion	1906 (45.0)	1882 (44.8)	−0.25	0.82	−0.80	0.82
Emergency visit	677 (16.0)	682 (16.2)	0.23	0.77	0.75	0.77
Avoidable emergency visit	115 (2.7)	103 (2.5)	−0.27	0.44	−0.86	0.44
*Outcomes captured at 1 year*						
Primary care change	2309 (54.6)	2302 (54.8)	0.22	0.84	0.72	0.84
Emergency visit	1279 (30.2)	1374 (32.7)	2.48	0.01	8.97	0.01
Avoidable emergency visit	241 (5.7)	257 (6.1)	0.42	0.41	1.36	0.41
*Preventive management*						
Lipid panel	1417 (33.5)	1443 (34.3)	0.86	0.41	2.76	0.41
Hemoglobin A1c	567 (13.4)	592 (14.1)	0.69	0.36	2.22	0.36
*Chronic illness diagnosis*						
Any illness	1176 (27.8)	1219 (29.0)	1.22	0.21	3.93	0.21
Asthma	192 (4.5)	221 (5.3)	0.72	0.12	2.33	0.12
COPD	16 (0.4)	13 (0.3)	−0.07	0.59	−0.22	0.59
Depression	375 (8.9)	409 (9.7)	0.87	0.17	2.81	0.17
Diabetes	353 (8.3)	364 (8.7)	0.32	0.60	1.03	0.60
Dyslipidemia	308 (7.3)	325 (7.7)	0.46	0.43	1.47	0.43
Hypertension	575 (13.6)	601 (14.3)	0.72	0.34	2.30	0.34
Ischemic heart disease	90 (2.1)	98 (2.3)	0.21	0.52	0.66	0.52

*COPD* chronic obstructive lung disease, *IHA* initial health assessment.

### Identification of chronic disease and preventive testing

At 1 year, identification of chronic disease was similar in the texting vs. control group: 29.0% vs. 27.8% (difference, 1.2%; 95% CI, −0.7 to 3.1%; *p* = 0.21; Table 2). The most commonly identified chronic diseases were hypertension (14.3% texting vs. 13.6% controls), depression (9.7% vs. 8.9%), and diabetes (8.7% vs. 8.3%). There was no difference in frequency of identification for any chronic disease between the two groups.

At 1 year, receipt of preventive testing was not different in the texting versus control group, including diabetes screening (14.1% vs. 13.4%; difference, 0.69%; 95% CI, −0.8% to 2.2%; *p* = 0.36) and cholesterol screening (34.3% vs. 33.5%; difference, −0.42%; 95% CI, −0.6 to1.4%; *p* = 0.41; Table 2).

### Emergency department utilization

At 120 days in the text message vs. control group, emergency department use at least once (16.2% vs. 16.0%; difference, 0.23%; 95% CI, −1.33 to 1.8%; *p* = 0.77) or for avoidable use (2.5% vs. 2.7% difference, −0.27%; 95% CI, −0.94 to 0.41%; *p* = 0.44) was not significantly different. At 1 year, use of the emergency department at least once was higher in the text message group (32.7% vs. 30.2%; difference, 2.5%; 95% CI, 0.5 to 4.5%; *p* = 0.014; Table 2). Avoidable emergency department use at least once (6.1% vs. 5.7%) was not statistically different (difference, 0.4%; 95% CI, −0.6 to 1.4%; *p* = 0.41).

### Instrumental variable analysis

The results were overall similar in our instrumental variable sensitivity analysis (Table 2). The magnitude of all effects was larger as estimated by instrumental variable analysis, but all estimated effects were statistically insignificant, with the exception of ED use, as found in the intention-to-treat analysis. In the instrumental variable analysis, the text messaging intervention increased ED use by 9.0% (*p* = 0.01) among patients who engaged with text messages and did not opt out.

## Discussion

Newly enrolled Medicaid beneficiaries who received tailored text messages encouraging them to visit primary care were not more likely to visit primary care within 120 days, receive preventive services, or obtain a diagnosis of a chronic disease. However, enrollees randomized to text messages were more likely to use the emergency department over a year.

There are several possible explanations for why text messages did not cause increased use of primary care. First, despite the support provided by the text messages, perhaps it was insufficient to surmount the barriers many beneficiaries face to make their first primary care appointment happen within 120 days^12,20^. For example, perhaps transportation to clinic or challenging work schedules prevented patients from receiving care. It is also possible that the text messages did trigger enrollees to schedule an appointment, but local PCPs had limited appointment availability. Or perhaps the text messaging intervention was not sufficiently personalized, for example able to suggest a local PCP with greater appointment availability. Second, text messages may be insufficient to drive younger adults who are not ill and do not perceive a need to visit a physician^21^.

Our work builds on and corroborates others. Prior work in a safety net population demonstrated that patients had access to and preferred text messages over e-mail, phone, and letters for communication^22^. While there are studies demonstrating text messages are effective, others have not found any benefit. For example, condition-specific text messages have had a limited effect^23–27^ and one study found text messages may be no better, except with regard to cost, than other outreach such as phone in reducing no-show rates^28^.

Multiple avenues may improve the efficacy of text messages. First, several groups have advocated following cultural relevancy principles for text messages^29,30^. This could also include involving patients and clinicians in the creation and usability testing of the messages. Second, text messages could be tied to financial incentives. For example, offering an incentive for participation linked to a loss aversion schema that requires a certain number of responses may lead to higher engagement^31^. Third, the text messages could be bundled with more support. Patients in a local health care delivery area could be asked about the major barriers they find when attempting to access primary care. If, for example, wait time or language concordance when attempting to schedule an appointment on the phone were barriers, instead of encouraging patients to schedule an appointment with primary care, the text message system could offer to schedule it for them.

One unexpected finding was that the intervention led to an increase in total ED visits at 1 year, but not at 120 days nor for avoidable ED admissions. What underlies this is unclear. Possibly the text messages reminded them of their insurance coverage in general, and when a health issue arose later in the year, this prompted an ED visit when other opportunities for care were not as easily available. This finding requires replication.

Our study has limitations. First, the intervention only had fair uptake, with about 31% of beneficiaries engaging in 1 text message response^32–34^. It is possible that with more uptake, results would have differed, though in our sensitivity analysis that addressed engagement we found no substantive differences. Second, we used administrative data, which does not contain rich sociodemographic data. It is possible that despite randomization, unobserved confounding existed between the two groups. In addition, these beneficiaries were new to the Medicaid plan, so we could not ascertain prior utilization or medical comorbidities. Third, about 30% disenrolled from the plan and 18% were excluded because of an invalid phone number. While this mirrors other Medicaid plan experiences, this could have confounded differences between the two groups^35^. Fourth, we analyzed text messages at 1 Medicaid managed care plan, limiting generalizability. Fifth, our analysis could not rule out supply-side constraints as driving our null finding, as discussed above.

Tailored text messages to new Medicaid beneficiaries were ineffective in increasing use of primary care within 120 days but increased ED utilization over 1 year.

## Methods

### Context and overview

Since 2008, to encourage more enrollees to obtain primary care, the California Department of Healthcare Services has required all Medicaid managed care plans to ensure that members undertake an initial health assessment (IHA) by their PCPs within 120 days of enrollment^36^. The IHA is a comprehensive assessment of physical and emotional health that catalogs appropriate preventive care and delineates a care plan that is done by a PCP during a visit. As a result of this mandate, the Medicaid managed care plan with which we partnered was interested in whether a low-cost text message platform could help newly enrolled beneficiaries engage in primary care within 120 days, with potential downstream benefits including use of preventive care, identification of chronic illness, and reducing emergency department utilization.

The Medicaid managed care plan collaborated with a private vendor specializing in text messages for health plans to offer tailored text messages to newly enrolled beneficiaries. The plan and vendor designed and implemented the randomized controlled trial. We performed a retrospective evaluation of their parallel-design, randomized controlled trial with participants randomly allocated to tailored text messages and traditional new member communication (intervention) versus traditional new member communication alone (control). Participants were enrolled between June 7, 2018 and August 7, 2018; follow-up ended July 30, 2019. The Medicaid plan did not have access to which enrollees were randomized to the intervention and the text message company did not have access to the outcome data. We independently merged and analyzed data from both entities.

The retrospective evaluation was approved by the Harvard Medical School Institutional Review Board as exempt. Because this was a quality improvement initiative of the Medicaid plan, users did not provide informed consent but did sign their standard beneficiary agreement and could opt out of text messages upon receipt of the first message. There was no contact with the users beyond the text messages.

### Setting and participants

Participants were all newly insured adult (≥18 years old) beneficiaries of a large, mostly urban, California Medicaid managed care health insurance plan who were newly enrolled during the above dates. The plan was only available to residents of select California counties^37^. Participants were excluded if they carried an “administrative label” given access would be different for this group, which occurred when a beneficiary had Medicaid with a share-of-cost requirement, resided in a long-term care facility, had other health insurance and Medicaid was only for secondary coverage, were enrolled under a special aid category, or were in hospice.

### Randomization

Each month, the Medicaid plan provided the text message company with the telephone numbers of newly enrolled beneficiaries. The text message company would scrub this list, excluding those without a valid cell phone number that accepts text messages (18% excluded). The text message company randomized remaining telephone numbers 1:1 to either receive their tailored text message program or receive usual care (no text messages). If two or more individuals were randomized in the same household to different arms, we retrospectively excluded all individuals in the household to prevent contamination (1.8% dropped individuals).

### Intervention

The tailored text messages were delivered during a 16-week program. Tailoring occurred around primary language, age, sex, and other proprietary algorithms. Participants received at least five messages per month. The system delivered information via text and was able to respond to participant free-text responses using natural language processing. During weeks 1–4, beneficiaries were welcomed, assessed for baseline knowledge regarding how to obtain primary care, and educated regarding the IHA. During weeks 5–14, beneficiaries were asked to schedule their first appointment with a PCP. They also received tailored messages on how to access a nurse advice line, behavioral health resources, and dental benefits. To encourage engagement with the text messaging service, they also received messages regarding wellness, air quality, and food insecurity. During weeks 15 and 16, their knowledge of how to get care was assessed. In both arms, the Medicaid plan sent its standard mailings to all new beneficiaries describing how to access benefits. Usual care received only these mailings. At any time, beneficiaries could cease participation.

### Outcomes and follow-up

All outcome data were obtained from the health plan’s administrative data or the text message company. Our primary outcome was completion of one or more primary care visits within 120 days of enrollment. A secondary outcome was receipt of the IHA within 120 days of enrollment. The norm is that this is completed at the primary care office, but some offices may not complete the IHA. We therefore report PCP IHA completion rates, which were similar in both groups (Supplementary Note). We examined engagement with text messages by noting to how many text messages a participant responded (with a response other than stop).

We also examined other outcomes that could be impacted by increased receipt of primary care. Our secondary outcomes included 1-year receipt of preventive care (hemoglobin A1c or lipid panel) and diagnosis of a chronic disease (asthma, chronic obstructive pulmonary disease, depression, diabetes, dyslipidemia, hypertension, or ischemic heart disease). We counted a single visit with relevant ICD-10 code as evidence of chronic disease. We also captured the rates of disenrollment with the health plan. Finally, we examined at both 120 days and 1 year the use of the emergency department and avoidable use of the emergency department (Supplementary Table 1).

### Statistical analysis

We present descriptive data on sociodemographics, engagement, and disenrollment with counts and percentages or means and 95% confidence intervals as appropriate. We first present differences in outcomes between all patients in the treatment and control group, also known as an intention-to-treat estimate. A standard intention to treat analysis estimates the effect of recommending a treatment, not the effect of the treatment on those who received it^38^. In this case, the intention-to-treat estimate is the estimated effect of being assigned to receive text messaging.

As a sensitivity analysis, we also used an instrumental variable analysis to address the limited engagement rate among patients to the text messaging intervention. Increasingly used in clinical trials, the instrumental variable analysis estimates the impact of the text messaging intervention among the patients who engaged^38,39^. We defined engagement as patients who responded to the text message at least once and did not opt out by saying “stop” at any point in the study period. The instrument was treatment assignment.

We performed all analyses with Stata version 15.1 (College Station, TX, USA). We considered two-sided *p*-values of < 0.05 to be significant.

### Reporting summary

Further information on experimental design is available in the Nature Research Reporting Summary linked to this paper.

## Supplementary information

Supplemental Material

Reporting Summary

## Data Availability

The data that support the findings of this study are available from health plan, but restrictions apply to the availability of these data, which were used under an agreement for the current study, and so are not publicly available. Data are however available from the authors upon reasonable request and with permission of the health plan.

## Code availability

The authors will make available upon reasonable request any previously unreported custom computer code or algorithm used to generate results that are reported in the paper and central to its main claims.
