# Supplementary material for: Randomized controlled study using text messages to help connect new medicaid beneficiaries to primary care
Source: NPJ Digit Med. 2021 Feb 15;4:26. doi: 10.1038/s41746-021-00389-5 (PMC7884833; doi:10.1038/s41746-021-00389-5)
Supplement: Supplementary file 1 — Supplemental Material [file 41746_2021_389_MOESM1_ESM.pdf]

Randomized Controlled Study Using Text Messages to Help Connect New Medicaid Beneficiaries to Primary Care

David M Levine

Pragya Kakani

Ateev Mehrotra

SUPPLEMENTARY MATERIALS

Supplementary Note. Initial Health Assessment completion rate by PCP.
Supplementary Table 1. Definition of avoidable use of the emergency department.
Supplementary Table 2. Baseline participant characteristics of engagers vs. non-engagers within texting arm

Supplementary Note. Initial Health Assessment completion rate by PCP.

One concern is that PCPs initially assigned to individual patients could be different in their Initial Health Assessment (IHA) completion rate, defined as the fraction of patients assigned to a given PCP who receive an IHA. To test this, we compared the IHA completion rate in both arms and found they were equivalent:

	Control (n=4231)	Texting (n=4201)
PCPs IHA completion rate, mean % (95% CI)	44.2 (43.7, 44.6)	44.3 (43.8, 44.7)

Of note, each PCP had an average number of 7.42 patients.

Supplementary Table 1. Definition of avoidable use of the emergency department.

Note: We were required to use the definition implemented by the Medicaid managed care company.

Diagnosis_Code	Diagnosis_Description
B35.4	Tinea corporis
B37.0	Candidal stomatitis
B37.2	Candidiasis of skin and nail
B37.3	Candidiasis of vulva and vagina
B37.41	Candidal cystitis and urethritis
B37.49	Other urogenital candidiasis
B37.7	Candidal sepsis
B37.81	Candidal esophagitis
B37.89	Other sites of candidiasis
B37.9	Candidiasis, unspecified
B55.2	Mucocutaneous leishmaniasis
B86	Scabies
B88.0	Other acariasis
B88.8	Other specified infestations
H10.013	Acute follicular conjunctivitis, bilateral
H10.021	Other mucopurulent conjunctivitis, right eye
H10.022	Other mucopurulent conjunctivitis, left eye
H10.023	Other mucopurulent conjunctivitis, bilateral
H10.10	Acute atopic conjunctivitis, unspecified eye
H10.11	Acute atopic conjunctivitis, right eye
H10.12	Acute atopic conjunctivitis, left eye
H10.13	Acute atopic conjunctivitis, bilateral
H10.232	Serous conjunctivitis, except viral, left eye
H10.30	Unspecified acute conjunctivitis, unspecified eye
H10.31	Unspecified acute conjunctivitis, right eye
H10.32	Unspecified acute conjunctivitis, left eye
H10.33	Unspecified acute conjunctivitis, bilateral
H10.401	Unspecified chronic conjunctivitis, right eye
H10.402	Unspecified chronic conjunctivitis, left eye
H10.403	Unspecified chronic conjunctivitis, bilateral
H10.421	Simple chronic conjunctivitis, right eye
H10.45	Other chronic allergic conjunctivitis

Diagnosis_Code	Diagnosis_Description
H10.533	Contact blepharoconjunctivitis, bilateral
H10.89	Other conjunctivitis
H10.9	Unspecified conjunctivitis
H66.001	Acute suppurative otitis media without spontaneous rupture of ear drum, right ear
H66.002	Acute suppurative otitis media without spontaneous rupture of ear drum, left ear
H66.003	Acute suppurative otitis media without spontaneous rupture of ear drum, bilateral
H66.004	Acute suppurative otitis media without spontaneous rupture of ear drum, recurrent, right ear
H66.005	Acute suppurative otitis media without spontaneous rupture of ear drum, recurrent, left ear
H66.006	Acute suppurative otitis media without spontaneous rupture of ear drum, recurrent, bilateral
H66.009	Acute suppurative otitis media without spontaneous rupture of ear drum, unspecified ear
H66.011	Acute suppurative otitis media with spontaneous rupture of ear drum,right ear
H66.012	Acute suppurative otitis media with spontaneous rupture of ear drum,left ear
H66.015	Acute suppurative otitis media with spontaneous rupture of ear drum,recurrent, left ear
H66.42	Suppurative otitis media, unspecified, left ear
H66.90	Otitis media, unspecified, unspecified ear
H66.91	Otitis media, unspecified, right ear
H66.92	Otitis media, unspecified, left ear
H66.93	Otitis media, unspecified, bilateral
J00	Acute nasopharyngitis [common cold]
J02.0	Streptococcal pharyngitis
J02.8	Acute pharyngitis due to other specified organisms
J02.9	Acute pharyngitis, unspecified
J06.0	Acute laryngopharyngitis
J06.9	Acute upper respiratory infection, unspecified
J20.0	Acute bronchitis due to Mycoplasma pneumoniae
J20.1	Acute bronchitis due to Hemophilus influenzae
J20.2	Acute bronchitis due to streptococcus
J20.4	Acute bronchitis due to parainfluenza virus
J20.6	Acute bronchitis due to rhinovirus

Diagnosis_Code	Diagnosis_Description
J20.8	Acute bronchitis due to other specified organisms
J20.9	Acute bronchitis, unspecified
J21.0	Acute bronchiolitis due to respiratory syncytial virus
J21.8	Acute bronchiolitis due to other specified organisms
J21.9	Acute bronchiolitis, unspecified
J31.0	Chronic rhinitis
J31.1	Chronic nasopharyngitis
J31.2	Chronic pharyngitis
J32.0	Chronic maxillary sinusitis
J32.1	Chronic frontal sinusitis
J32.2	Chronic ethmoidal sinusitis
J32.3	Chronic sphenoidal sinusitis
J32.8	Other chronic sinusitis
J32.9	Chronic sinusitis, unspecified
J35.01	Chronic tonsillitis
J35.02	Chronic adenoiditis
J35.03	Chronic tonsillitis and adenoiditis
J35.1	Hypertrophy of tonsils
J35.2	Hypertrophy of adenoids
J35.3	Hypertrophy of tonsils with hypertrophy of adenoids
J35.8	Other chronic diseases of tonsils and adenoids
L29.0	Pruritus ani
L29.2	Pruritus vulvae
L29.3	Anogenital pruritus, unspecified
L29.8	Other pruritus
L29.9	Pruritus, unspecified
L71.9	Rosacea, unspecified
L74.0	Miliaria rubra
L74.3	Miliaria, unspecified
M53.3	Sacrococcygeal disorders, not elsewhere classified
M54.40	Lumbago with sciatica, unspecified side
M54.41	Lumbago with sciatica, right side
M54.42	Lumbago with sciatica, left side
M54.5	Low back pain
M54.9	Dorsalgia, unspecified
N30.00	Acute cystitis without hematuria

Diagnosis_Code	Diagnosis_Description
N30.01	Acute cystitis with hematuria
N30.10	Interstitial cystitis (chronic) without hematuria
N30.30	Trigonitis without hematuria
N30.80	Other cystitis without hematuria
N30.90	Cystitis, unspecified without hematuria
N30.91	Cystitis, unspecified with hematuria
N39.0	Urinary tract infection, site not specified
N72	Inflammatory disease of cervix uteri
N76.0	Acute vaginitis
N76.1	Subacute and chronic vaginitis
N76.2	Acute vulvitis
N97.0	Female infertility associated with anovulation
R29.898	Other symptoms and signs involving the musculoskeletal system
R51	Headache
Z00.00	Encounter for general adult medical examination without abnormal findings
Z00.01	Encounter for general adult medical examination with abnormal findings
Z00.110	Health examination for newborn under 8 days old
Z00.111	Health examination for newborn 8 to 28 days old
Z00.121	Encounter for routine child health examination with abnormal findings
Z00.129	Encounter for routine child health examination without abnormal findings
Z00.3	Encounter for examination for adolescent development state
Z00.8	Encounter for other general examination
Z01.00	Encounter for examination of eyes and vision without abnormal findings
Z01.810	Encounter for preprocedural cardiovascular examination
Z01.811	Encounter for preprocedural respiratory examination
Z01.812	Encounter for preprocedural laboratory examination
Z01.818	Encounter for other preprocedural examination
Z01.89	Encounter for other specified special examinations
Z02.5	Encounter for examination for participation in sport
Z02.79	Encounter for issue of other medical certificate
Z02.83	Encounter for blood-alcohol and blood-drug test
Z02.89	Encounter for other administrative examinations
Z02.9	Encounter for administrative examinations, unspecified
Z04.6	Encounter for general psychiatric examination, requested by authority
Z04.8	Encounter for examination and observation for other specified reasons

Diagnosis_Code	Diagnosis_Description
Z09	Encounter for follow-up examination after completed treatment for conditions other than malignant neoplasm
Z32.00	Encounter for pregnancy test, result unknown
Z32.01	Encounter for pregnancy test, result positive
Z32.02	Encounter for pregnancy test, result negative
Z76.0	Encounter for issue of repeat prescription

Supplementary Table 2. Baseline participant characteristics of engagers vs. non-engagers within texting arm

	Engagers (n=1305)	Non-engagers (n=2896)
Age, mean (95% CI)	38.4 (37.7, 39.2)	36.6 (36.1, 37.1)
Female, n (%)[a]	757 (58.1)	1569 (54.2)
English is first language, n (%)	1168 (89.5%)	2573 (88.9%)
Race/ethnicity, n (%)		
White	312 (23.9%)	695 (24.0%)
Black	122 (9.3%)	295 (10.2%)
Hispanic	583(44.7%)	1413 (48.8%)
Other	122 (9.3%)	203 (7.0%)
Unknown	166 (12.7%)	290 (10.0%)

[a]: 1 person in texting arm was missing data on gender
