# Supplementary material for: Randomized controlled study using text messages to help connect new medicaid beneficiaries to primary care
Source: NPJ Digit Med. 2021 Feb 15;4:26. doi: 10.1038/s41746-021-00389-5 (PMC7884833; doi:10.1038/s41746-021-00389-5)
Supplement: Supplementary file 2 — Reporting Summary [file 41746_2021_389_MOESM2_ESM.pdf]

nature research

Corresponding author(s): David Levine

Last updated by author(s): Dec 13, 2020

Reporting Summary

Nature Research wishes to improve the reproducibility of the work that we publish. This form provides structure for consistency and transparency in reporting. For further information on Nature Research policies, see our Editorial Policies and the Editorial Policy Checklist.

Statistics

For all statistical analyses, confirm that the following items are present in the figure legend, table legend, main text, or Methods section.

n/a	Confirmed	
☐	☒	The exact sample size (n) for each experimental group/condition, given as a discrete number and unit of measurement
☐	☒	A statement on whether measurements were taken from distinct samples or whether the same sample was measured repeatedly
☐	☒	The statistical test(s) used AND whether they are one- or two-sided<br>*Only common tests should be described solely by name; describe more complex techniques in the Methods section.*
☐	☒	A description of all covariates tested
☐	☒	A description of any assumptions or corrections, such as tests of normality and adjustment for multiple comparisons
☐	☒	A full description of the statistical parameters including central tendency (e.g. means) or other basic estimates (e.g. regression coefficient) AND variation (e.g. standard deviation) or associated estimates of uncertainty (e.g. confidence intervals)
☐	☒	For null hypothesis testing, the test statistic (e.g. F, t, r) with confidence intervals, effect sizes, degrees of freedom and P value noted<br>*Give P values as exact values whenever suitable.*
☒	☐	For Bayesian analysis, information on the choice of priors and Markov chain Monte Carlo settings
☒	☐	For hierarchical and complex designs, identification of the appropriate level for tests and full reporting of outcomes
☒	☐	Estimates of effect sizes (e.g. Cohen's d, Pearson's r), indicating how they were calculated

Our web collection on statistics for biologists contains articles on many of the points above.

Software and code

Policy information about availability of computer code

Data collection	Proprietary custom code used to collect data.
Data analysis	Tailored STATA version 15.1 used to analyze data.

For manuscripts utilizing custom algorithms or software that are central to the research but not yet described in published literature, software must be made available to editors and reviewers. We strongly encourage code deposition in a community repository (e.g. GitHub). See the Nature Research guidelines for submitting code & software for further information.

Data

Policy information about availability of data

All manuscripts must include a data availability statement. This statement should provide the following information, where applicable:

- Accession codes, unique identifiers, or web links for publicly available datasets
- A list of figures that have associated raw data
- A description of any restrictions on data availability

The data that support the findings of this study are available from health plan, but restrictions apply to the availability of these data, which were used under an agreement for the current study, and so are not publicly available. Data are however available from the authors upon reasonable request and with permission of the health plan.

Field-specific reporting

Please select the one below that is the best fit for your research. If you are not sure, read the appropriate sections before making your selection.

☐ Life sciences ☒ Behavioural & social sciences ☐ Ecological, evolutionary & environmental sciences

For a reference copy of the document with all sections, see nature.com/documents/nr-reporting-summary-flat.pdf

Behavioural & social sciences study design

All studies must disclose on these points even when the disclosure is negative.

Study description	Randomized controlled trial
Research sample	All new beneficiaries to a large Medicaid managed care plan were included June 7, 2018 and August 7, 2018.
Sampling strategy	All new beneficiaries were randomized to receive text messages or usual care. This approach was governed by the feasibility of maintaining operations in a large insurance plan for longer and the real-world nature of this evaluation.
Data collection	The insurance plan collected and summarized data from its usual administrative claims database. The text messaging company collected and summarized data from its usual database. Both entities were blinded to one-another's data.
Timing	June 7, 2018 - July 30, 2019
Data exclusions	Participants were excluded if they carried an “administrative label” given access would be different for this group, which occurred when a beneficiary had Medicaid with a share-of-cost requirement, resided in a long-term care facility, had other health insurance and Medicaid was only for secondary coverage, were enrolled under a special aid category, or were in hospice.
Non-participation	31% of beneficiaries engaging in more than 1 text message response.
Randomization	1:1 simple random sample

Reporting for specific materials, systems and methods

We require information from authors about some types of materials, experimental systems and methods used in many studies. Here, indicate whether each material, system or method listed is relevant to your study. If you are not sure if a list item applies to your research, read the appropriate section before selecting a response.

Materials & experimental systems

n/a	Involved in the study
☒	☐ Antibodies
☒	☐ Eukaryotic cell lines
☒	☐ Palaeontology and archaeology
☒	☐ Animals and other organisms
☒	☐ Human research participants
☒	☐ Clinical data
☒	☐ Dual use research of concern

Methods

n/a	Involved in the study
☒	☐ ChIP-seq
☒	☐ Flow cytometry
☒	☐ MRI-based neuroimaging
